# Hypoxic Tumor-Derived Exosomal miR-199a-3p Promote Gastric Cancer Metastasis via MAP3K4

**DOI:** 10.7150/jca.83909

**Published:** 2023-07-15

**Authors:** Li Li, Lei Wang, Jia-li Yang, Hui-Ju Wang, Yuan-yu Wang

**Affiliations:** 1 General Surgery, Cancer Center, Department of Gastrointestinal and Pancreatic Surgery, Zhejiang Provincial People's Hospital (Affiliated People's Hospital, Hangzhou Medical College), Hangzhou, Zhejiang 310014, China.; 2 Key Laboratory of Gastroenterology of Zhejiang Province, Zhejiang Provincial People's Hospital, Affiliated People's Hospital, Hangzhou Medical College, Hangzhou, Zhejiang, China.; 3 Department of Gastrointestinal Surgery, Central Hospital Affiliated to Shandong First Medical University, Jinan 250013, China.; 4 Zhejiang Chinese Medical University, Hangzhou, Zhejiang 310053, China.

**Keywords:** Hypoxic, Tumor-Derived Exosomal, miR-199a-3p, Gastric Cancer, Metastasis, MAP3K4

## Abstract

**Background:** Accumulating evidence has suggested the significant role of hypoxic tumor-derived exosomal miRNA in regulating gastric cancer metastasis, while its regulatory mechanism in gastric cancer remains unelucidated.

**Methods:** AGS and HGC-27 cell were cultured in hypoxia, and the expression of exosome miR-199a-3p was extracted and detected. Label free proteomic analysis, bioinformatics analysis, biluciferase assay and Westblot were used to verify the signaling pathway and target genes regulated by miR-199a-3p. Invasion and migration experiments were conducted to verify whether exosome miR-199a-3p can promote tumor growth and metastasis under hypoxia. Immunohistochemistry was used to detect the expression of MAP3K4 in gastric cancer.

**Results:** We first demonstrated that expression of tumor-derived exosomal miR-199a-3p is upregulated in AGS and HGC-27 cells in hypoxic conditions and tumor-derived exosomal miR-199a-3p promoted invasion and metastasis, which was evidenced by invasion and migration assays. Mechanistically, we validated that miR-199a-3p may act as a oncogene by modulating the expression of its downstream molecule MAP3K4.

**Conclusion:** Our results demonstrate that tumor-derived exosomal miR-199a-3p promotes invasion and metastasis in hypoxic conditions and the MAP3K4 signal axis may serve as a therapeutic target for gastric cancer.

## Introduction

Gastric cancer has the fifth highest incidence and third highest mortality of malignant tumors worldwide, gastric cancers in China accounting for 44.1% and 49.9% of incidence and mortality, respectively, worldwide 1. Furthermore, gastric cancer has the second and third highest incidence of malignant tumors in Chinese men and women, respectively, and the third highest mortality for both 2. Radical resection plus lymph node dissection has long been the preferred treatment for gastric cancer. With developments in surgical techniques, chemoradiotherapy and targeted therapy, the survival rate of patients with gastric cancer has gradually increased. The 5-year survival rates in South Korea and Japan are as high as 68.9% and 60.3%, respectively, whereas the rate is very low in China (35.9%) 3.

Exosomes, which are naturally formed vesicles, play an important role in tumor formation and progression. Their secretion, composition and function are impacted by the tumor microenvironment. Exosomes secreted by hypoxic cells impact tumor development, growth, angiogenesis and progression through multiple signaling pathways 4. Hypoxia-induced exosomal miRNA promotes tumor proliferation, invasion and metastasis 5-6. In a previous study, miR-199a-3p was found to be strongly expressed in gastric cancer tissue and to promote invasion and metastasis of gastric cancer cells, thus impacting prognosis 7. The clinic applications and potential mechanisms of miR-199a-3p on a wide variety of human malignancie had been extensively investigated and clearly elucidated in a great number of previous studies. This study mainly investigated the effects and potential mechanisms hypoxic tumor-derived exosomal miR-199a-3p promoting gastric cancer metastasis.

## Materials and methods

### Cell culture and hypoxia

A normal gastric cell line (GES-1) and gastric cancer cell lines AGS and HGC-27 (purchased from Shanghai Fuheng Biotechnology, Shanghai, China) were cultured with RRMI-1640 medium containing 10% fetal bovine serum. When the cell density reached 70%-80%, 15 mL exosome serum-free medium (UmiBio, Shanghai, China) was added to each dish of the normal culture (NC) group, cultured for 24 h, and the supernatant reserved. In the hypoxia group, 150 μmol/L CoCl2 was added to an exosome serum-free medium to simulate hypoxia and the supernatant collected after the cells had been cultured for 24 and 48 h.

### Exosome extraction and transmission electron microscopy (TEM)

Exosomes were extracted by ultracentrifugation, suspended in PBS and stored at -80°C until use. The frozen exosomes were gathered, melted, mixed with an equal volume of 4% PFA, centrifuged at 100,000 g for 2 h, resuspended, centrifuged again at 100,000 g, and precipitated to 50-100 µL 2% PFA. Next, 5 µL exosome suspension was added on a Formvar-carbon supported copper grid and photographed by TEM at 80 kV.

### Exosome RNA extraction and quality testing

After thawing, the exosomes were centrifuged at 12,000 g at 4°C for 10 min and a 250 µL sample transferred to a 1.5 mL centrifuge tube. Exosomal RNA was extracted using TRIzol reagent (Invitrogen Life Technologies) and the concentration and purity determined by using NanoDrop® ND-1000.

### Label-free proteomics analysis

Exosomes were determined by liquid chromatography with tandem mass spectrometry after precipitation, trypsin enzymolysis, polypeptide desalination, and resolubilization by Buffer A to 10 µL (Kangchen Biotech, Shanghai, China) 8. MaxQuant (Version 2.0.1.0) was used for database searching and quantitative analysis of raw data obtained by liquid chromatography with tandem mass spectrometry. The protein database was UNIPROT_HUMAN_2021_02; quantitative method: MS1 quantification.

### Reverse transcription polymerase chain reaction of exosomal RNA

RNA reverse transcription was performed by using a reverse transcription kit (Arraystar; Rockville, Maryland, USA), and RT-PCR was performed by using SYBR Green PCR Master Mix (Applied Biosystems). The following primers were provided by Shanghai Jierui Bioengineering (Shanghai, China), U6: 5′CGCTTCACGAATTTGCGTGTCAT3′, miR-199a-3p: 5'GTCGTATCCAGTGCGTGTCGTGGAGTCGGCAATTGCACTGGATACGACTAACCA3′. Data were analyzed by 2-△△CT method, ΔCt=Ct(miR-199a-3p)-Ct(U6).

### Firefly and Renilla luciferases

AGS and HGC-27 cells were cultured in 96-well plates. DMEM (10 μL) was mixed with 0.16 μg H-MAP3K4-3 'UTR target plasmid and 5 pmol miR-199a-3p/negative control, and placed at room temperature (Solution A). Next, 10 μL DMEM was thoroughly mixed with 0.3 μL transfection reagent (0.8 mg/mL; Hanheng Medical Technology) (Solution B), and placed at room temperature for 5 min. Solutions A and B were then mixed thoroughly and stored at room temperature for 20 min. The medium was replaced by a fresh medium before transfection, after which the transfection mixture was added and mixed. The cells were then incubated at 37°C in 5% CO2. The medium was replaced by fresh medium after 6 h of transfection and cells harvested after 48 h of transfection, followed by dual luciferase gene reporter assay (Promega Dual-luciferase System; Promega,…).

### Invasion and migration assays

GES-1, AGS and HGC-27 were cultured with RPMI-1640 containing 10% fetal bovine serum. When the cell density had reached 70%-80%, the cells were digested and harvested by trypsin, rinsed with serum-free medium three times, counted to adjust cell concentration, and seeded at 5×104 cells/well into a migration chamber and 2×105 cells/well into the upper chamber of an invasion chamber. Complete medium containing serum was added to the lower chamber. After 24 h of culture, the migration and invasion chambers were removed and the cells in the upper chamber swabbed. After methanol fixation and Giemsa staining, the cells that had passed through the membrane were counted under microscopy.

### Western blotting

AGS and HGC-27 cells were cultured and transfected with miR-199a-3p inhibitor. After 24 h, the gastric cancer cell line was harvested for western blotting to assess expression of MAP3K4 (MAP3K4 antibody; concentration 1:1000; Affinity, USA)).

### Paraffin-embedded gastric cancer tissue

Relevant data on 436 patients with gastric cancer were collected from January 1998 to December 2004 in our hospital. All diagnoses had been pathologically confirmed and the patients had not undergone preoperative chemoradiotherapy. The study cohort comprised 311 men and 125 women, making the male-to-female ratio 2.49:1, and the median age was 60 years (range, 17-91 years). All patients were followed up for more than 5 years from January 1998 to December 2008. Survival was calculated from the date of surgery to the end of follow-up or the date of death from recurrence or metastasis. All cancer patients received routine chemotherapy after surgery and no radiation. The Review Board of our Hospital Ethics Committee approved the study, and informed consent was obtained from each participant before data collection. All experiments were performed in accordance with relevant guidelines and regulations of our Hospital Ethics Committee.

### Immunohistochemical staining and evaluation

Immunohistochemical staining was performed in accordance with the instructions accompanying MAP3K4 kits (1:500; Santa Cruz Biotechnology, Dallas, TX, USA) 9. The stained sections were assessed under light microscopy (Nikon, Tokyo, Japan) by two independent pathologists. Representative images were obtained. The scoring scale for degree of positivity was as follows: 0 (≤5%), 1 (6%-25%), 2 (26%-50%), and 3 (> 51%) 9. Intensity scores were: 0 (no staining), 1 (weak staining), 2 (medium staining), and 3 (strong staining). Finally, the scores for degree of positivity were multiplied by the intensity scores. Staining index scores ≥ 4 and <4 were defined as strong and weak MAP3K4 expression, respectively.

## Results

### Transmission electron microscopy

**Figure 1:**
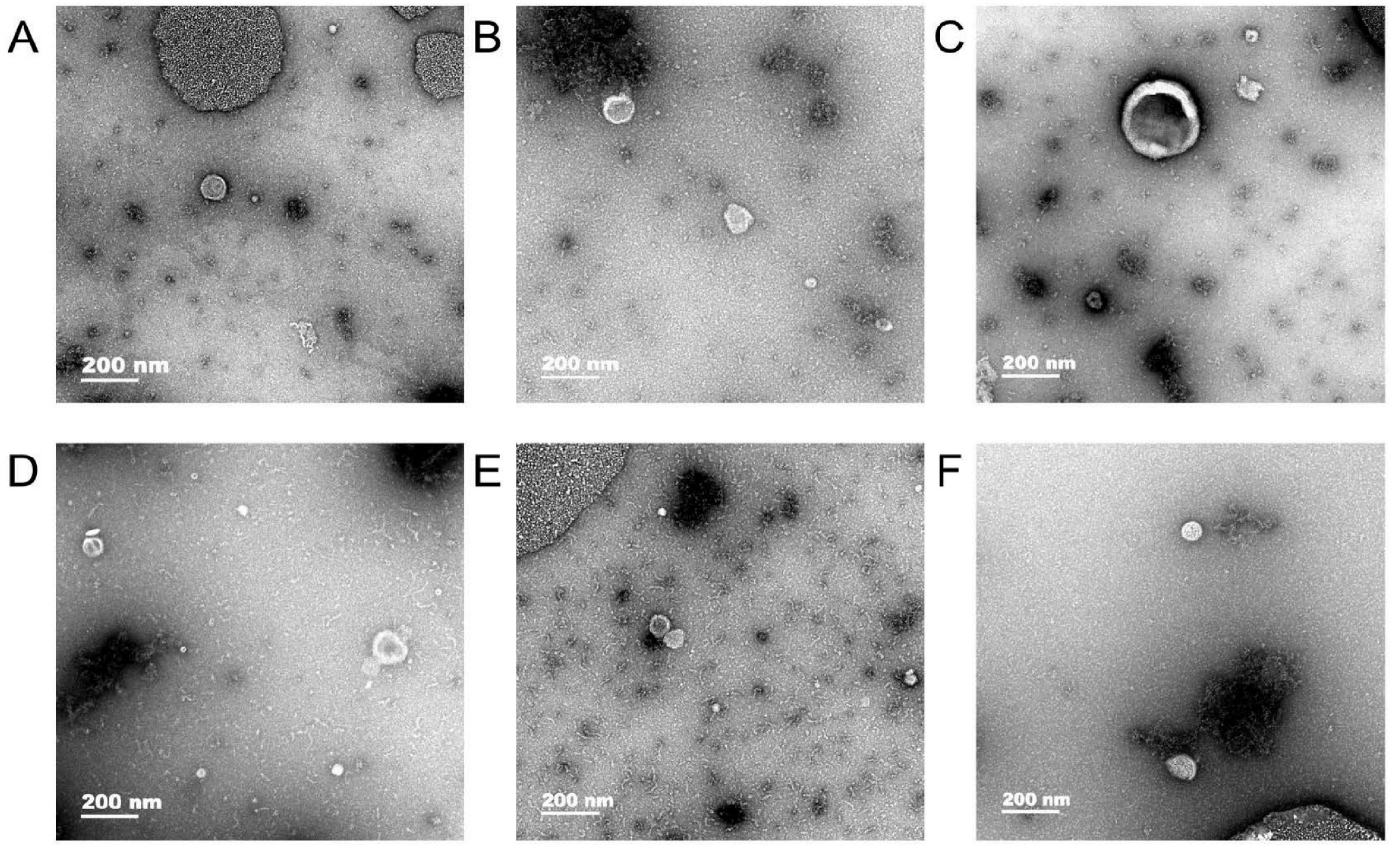
Transmission electron microscopy. In human gastric cell line GES-1 and gastric cancer cell lines AGS and HGC-27 under normal and hypoxic conditions, the diameter of exosomes were approximately 50-100 nm. A: The exosomes of GES-1 in normoxia, B: The exosomes of GES-1 in hypoxia, C: The exosomes of AGS in normoxia, D: The exosomes of HGC-27 in hypoxia, E: The exosomes of HGC-27 in normoxia, F: The exosomes of HGC-27 in hypoxia.

Exosomes were observed in human gastric cell line GES-1 and gastric cancer cell lines AGS and HGC-27 under normal and hypoxic conditions and the diameter of exosomes were approximately 50-100 nm (Fig. 1).

### Detection of exosomal RNA quality

RNA purity was determined using NanoDrop® ND-1000. The A260/A280 ratio of the RNA solutions was in the range of 1.8 to 2.1.

### Label-free proteomics analysis

**Figure 2:**
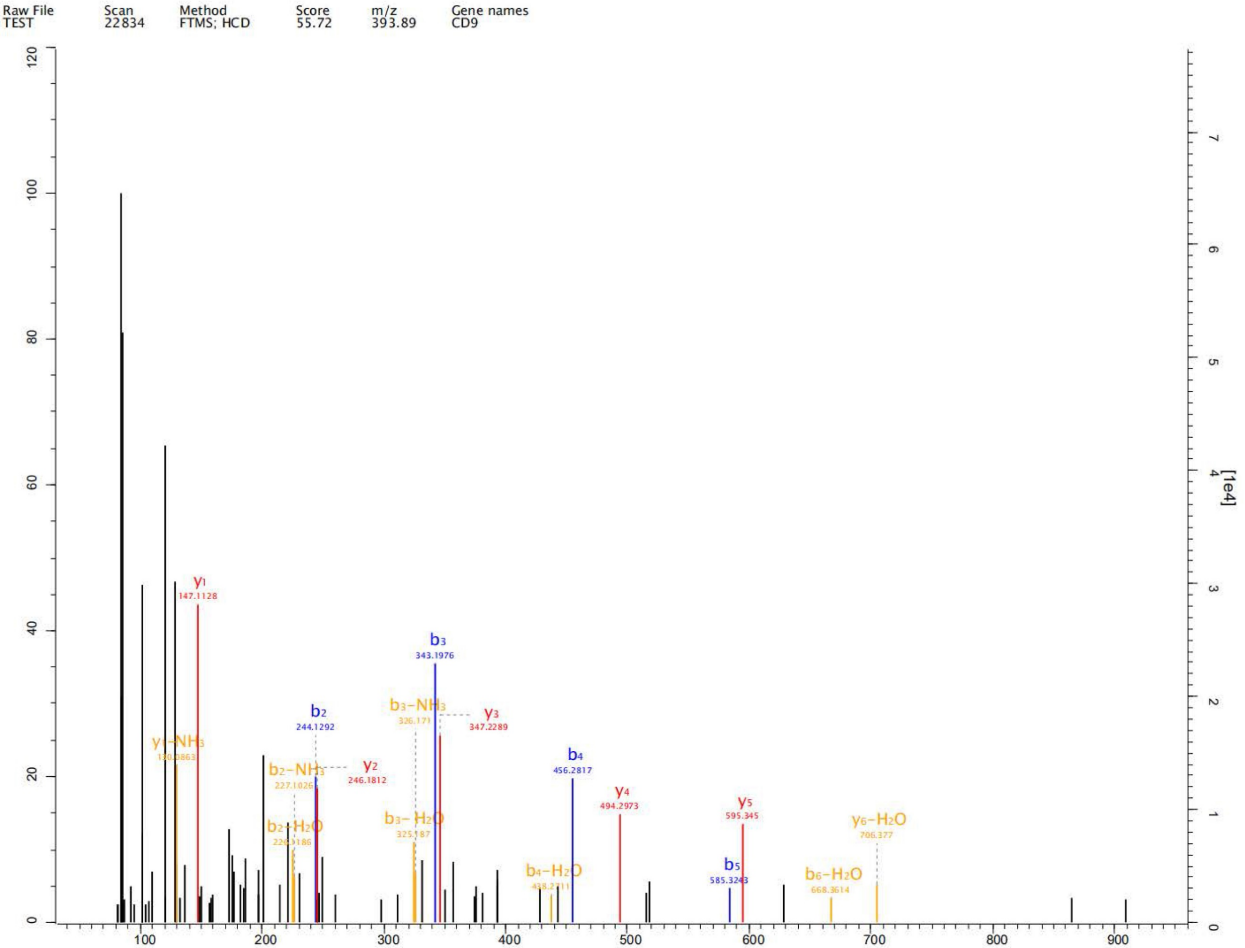
Expression of exosome markers CD9 was detected in the extracted exosomes by label-free proteomics analysis.

**Figure 3:**
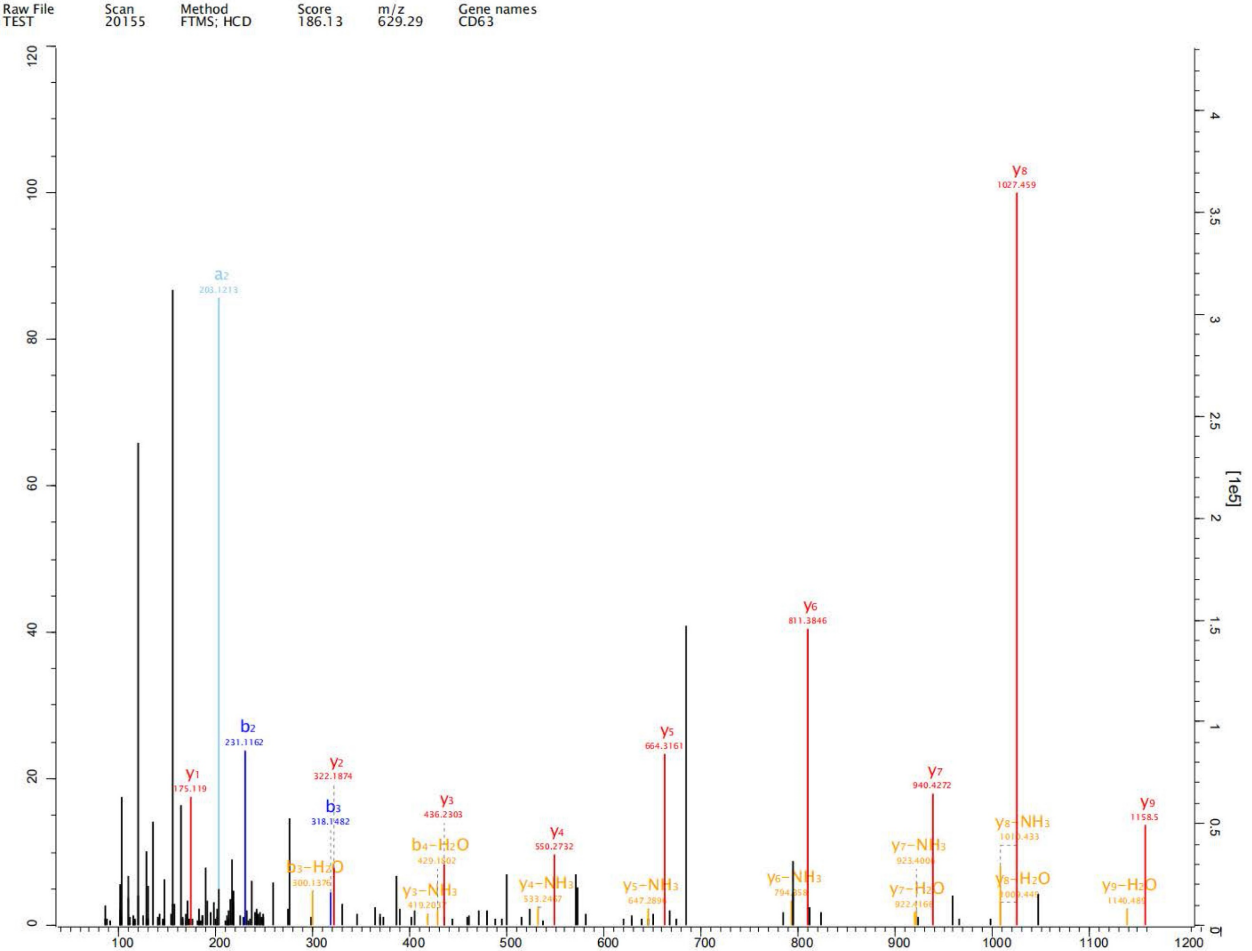
Expression of exosome markers CD63 was detected in the extracted exosomes by label-free proteomics analysis.

**Figure 4:**
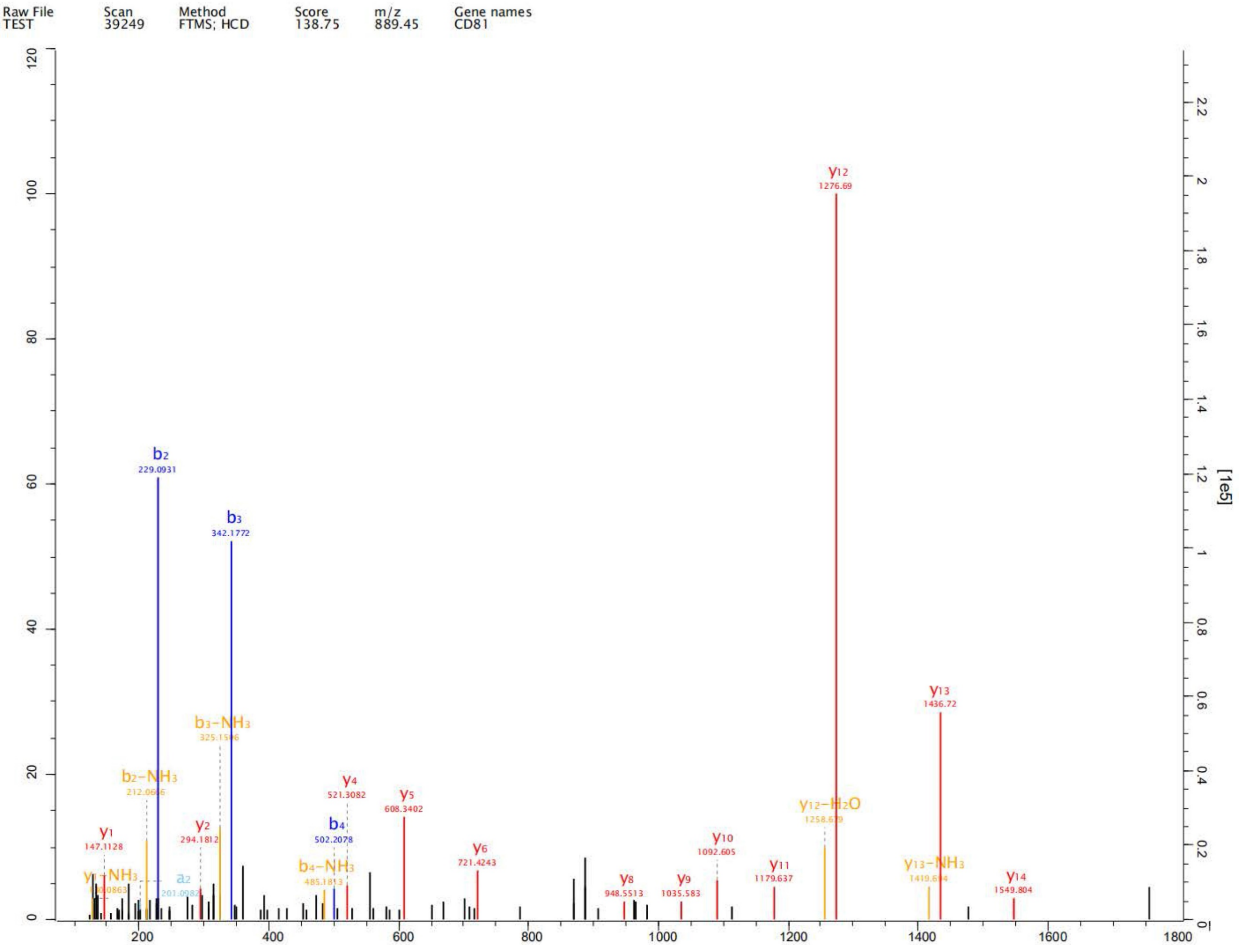
Expression of exosome markers CD81 was detected in the extracted exosomes by label-free proteomics analysis.

Expression of exosome markers CD9, CD63 and CD81 was detected in the extracted exosomes by label-free proteomics analysis, proving that we had extracted exosomal components (Fig. 2-4).

Through label-free proteomics analysis, the screened proteins were analyzed for functional classification and signal pathway. It was found that the selected proteins involved regulation of the actin cytoskeleton and the PI3K-Akt, ErbB, MAPK, sphingolipid, and AMPK signaling pathways, among others.

**Figure 5:**
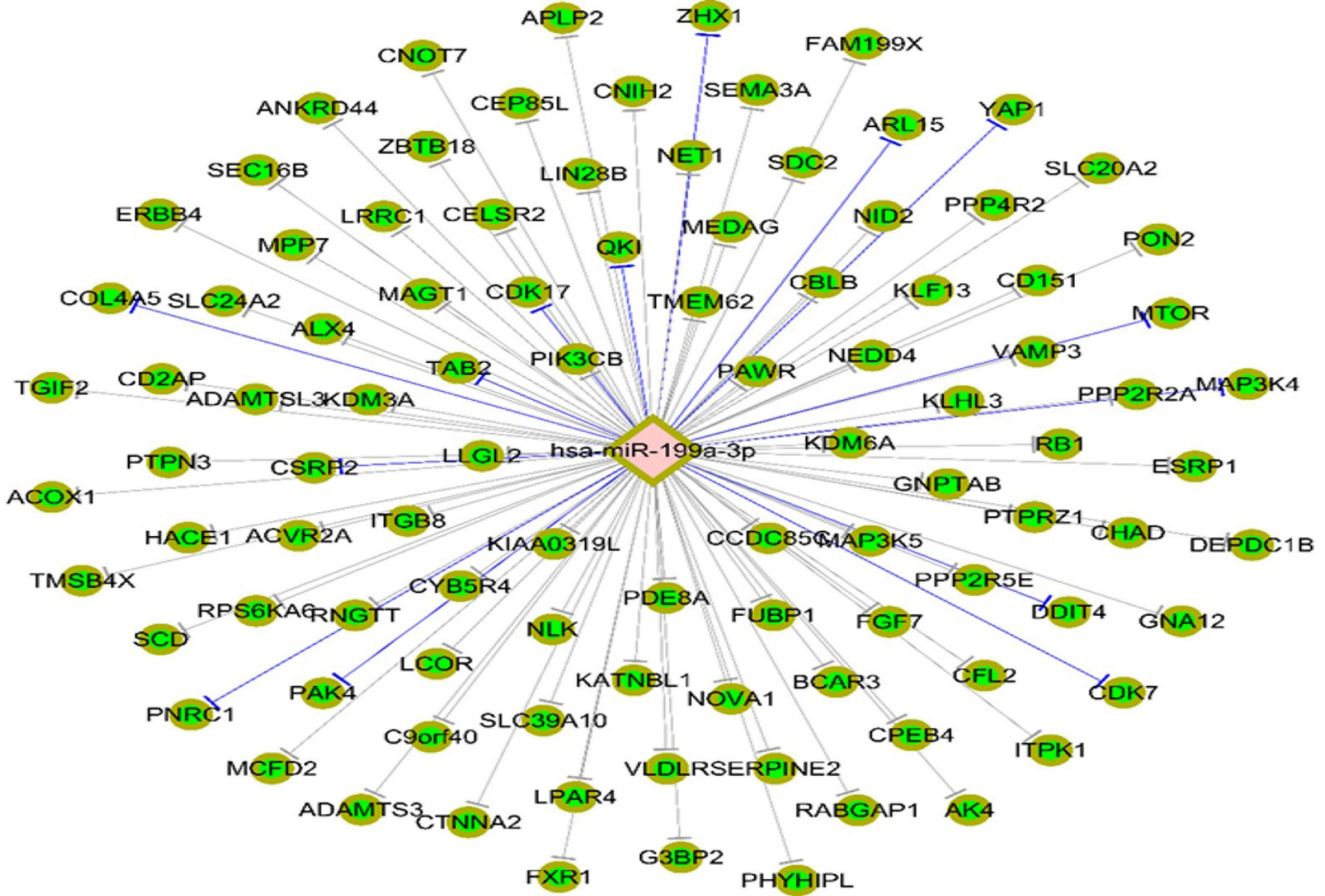
Potential target genes of miR-199a-3p were predicted using Targetscan7.1 and revealing that 146 genes, including *MAP3K4*.

Targetscan7.1 (http://www.targetscan.org/vert_71/) was used to predict the mRNA that could be combined with miR-199a-3p, revealing that 146 genes, including MAP3K4, are potential target genes of miR-199a-3p (Fig. 5).

### Expression of miR-199a-3p in exosomes of GES-1, AGS and HGC-27 under normal and hypoxic states

Expression of miR-199a-3p in AGS and HGC-27 cells in hypoxic conditions is significantly stronger than that in normal conditions. In contrast we found no difference between the two states in expression in GES-1 cells.

### Firefly and Renilla luciferases

**Figure 6:**
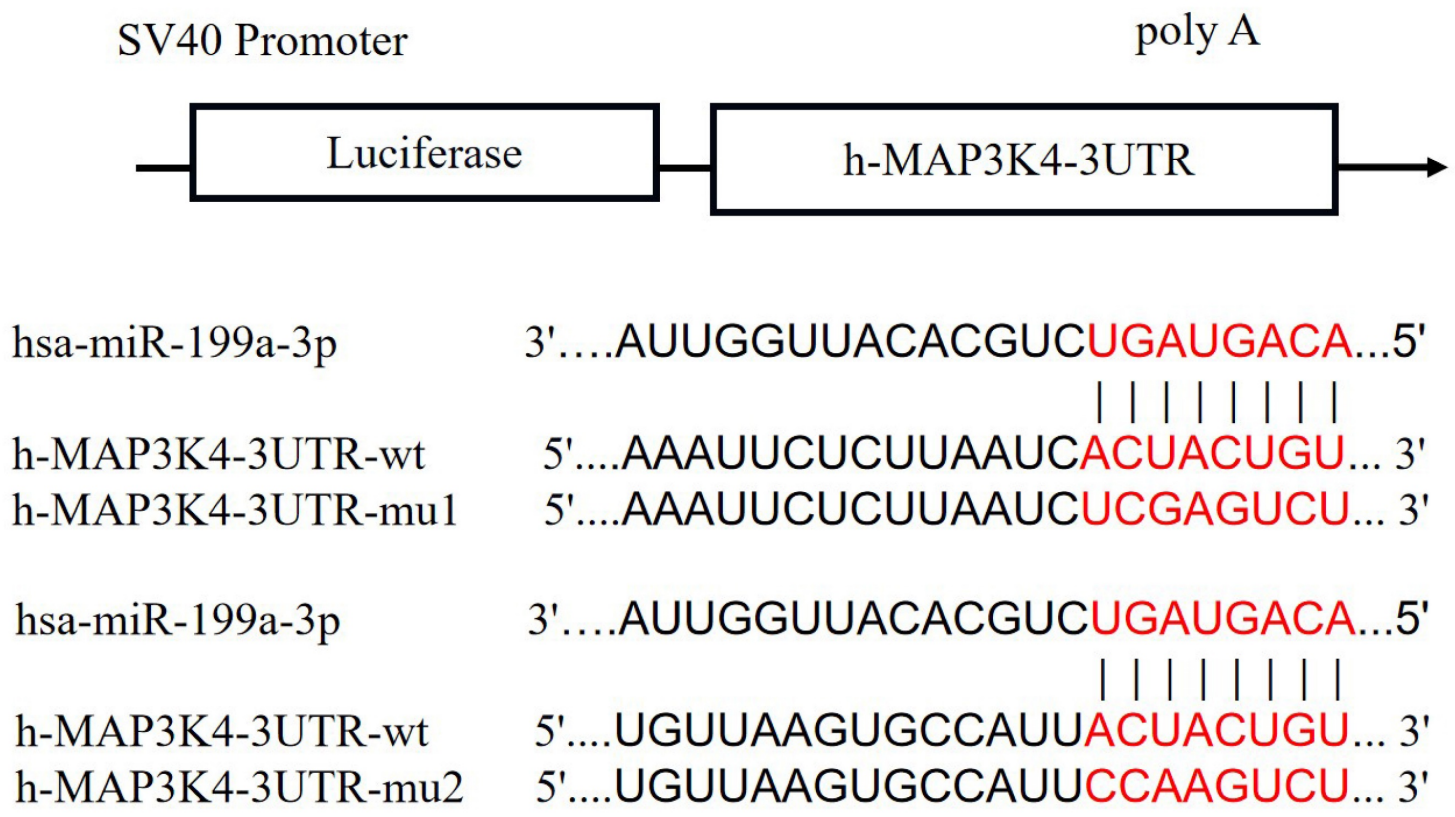
Schematic diagram of miR-199a-3p binding to MAP3K4-3UTR target sites.

**Figure 7:**
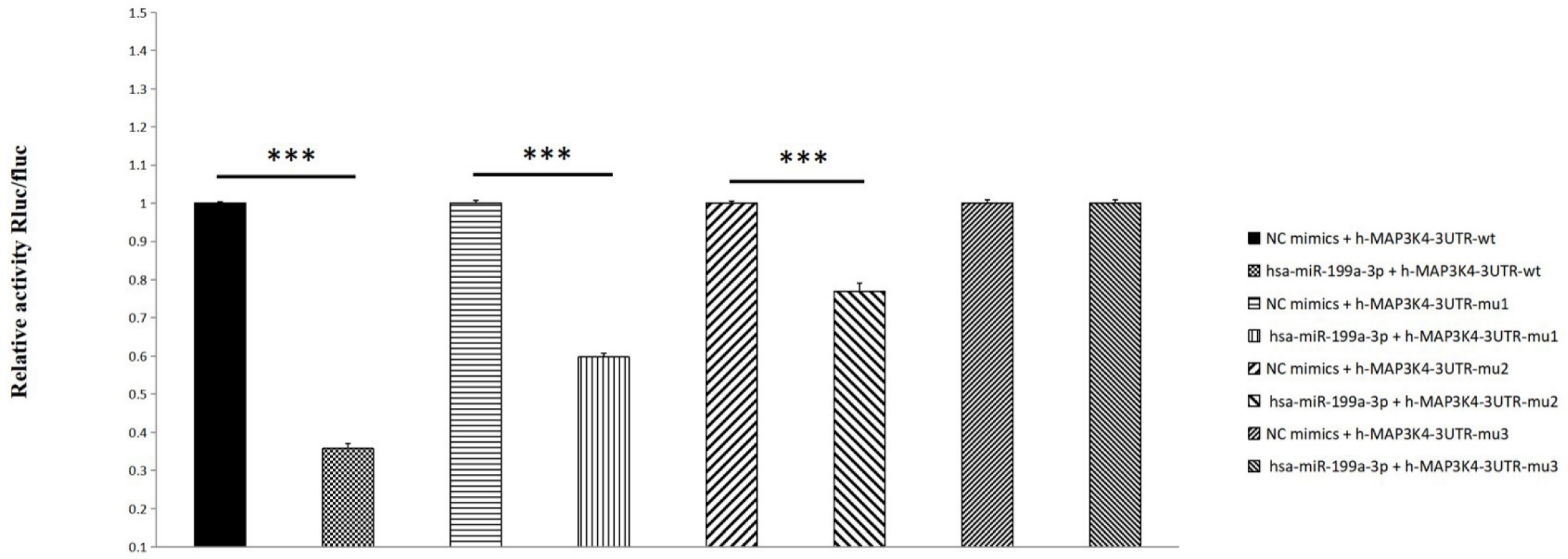
Dual luciferase reporter assay was used to detect the interaction between HSA-Mir-199a-3p and H-MAP3K4-3UTR.

Compared with the NC group, miR-199a-3p significantly down-regulates luciferase expression of h-MAP3K4-3UTR-WT (P < 0.001), indicating a binding effect between them (Fig. 6). Mir-199a-3p significantly down-regulates luciferase expression of h-MAP3K4-3UTR-MUT1 and h-MAP3K4-3UTR-MUT2 after mutation (P < 0.001), whereas miR-199a-3p does not down-regulate luciferase expression of h-MAP3K4-3UTR-MUT3 (P > 0.05), indicating that MUT1 and MUT2 are both binding sites for miR-199a-3p and h-MAP3K4-3UTR-WT (Fig. 7).

### Western blotting

**Figure 8:**
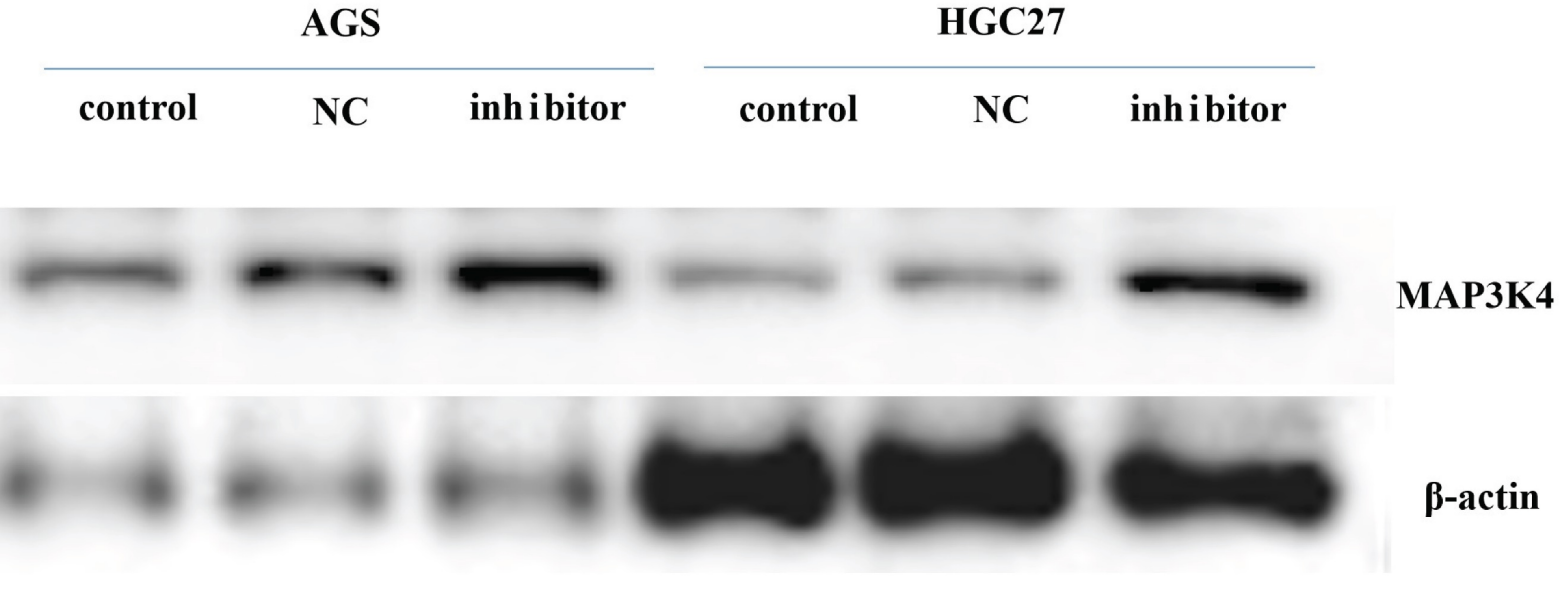
The expression of MAP3K4 increased 24 h after transfection with miR-199a-3p inhibitor in gastric cancer cell line.

Twenty-four h after transfection with miR-199a-3p inhibitor, the gastric cancer cell line was harvested for western blotting to assess expression of MAP3K4 (MAP3K4 antibody-n-terminal-AF0818 (Affinity) * 100 μL 1:1000). It was found that expression of MAP3K4 increased 24 h after transfection with miR-199a-3p inhibitor (Fig. 8).

### Migration assay

**Figure 9:**
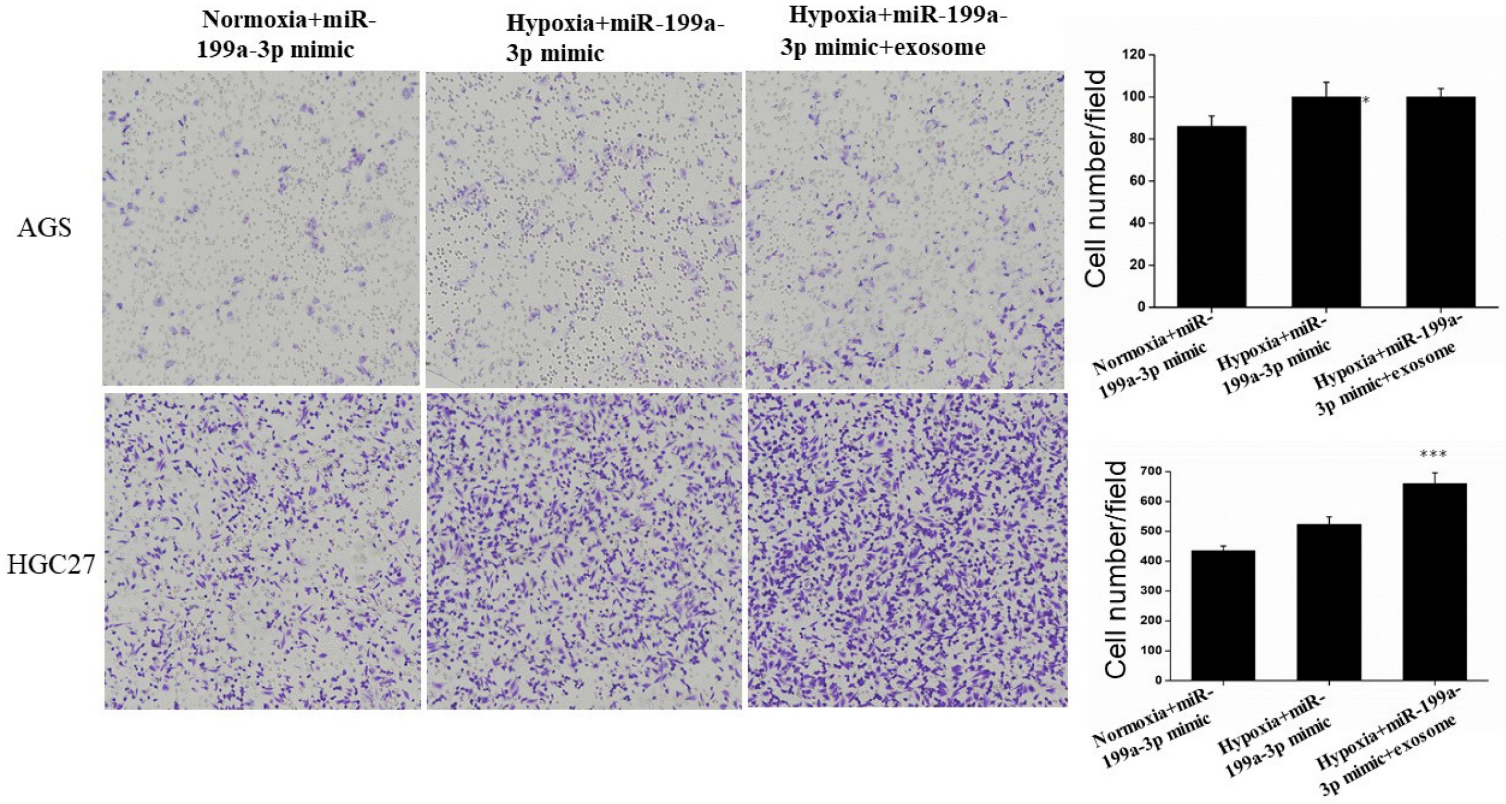
Transfection of exosomes and miR-199a-3p under hypoxic conditions also enhances the ability of AGS and HGC27 cells to migrate.

Under hypoxic conditions, miR-199a-3p promotes the ability of AGS and HGC27 cells to migrate. Transfection of exosomes and miR-199a-3p under hypoxic conditions also enhances the ability of tumor cells to migrate, hypoxic conditions more strongly promoting migration of HGC27 cells (Fig. 9).

### Invasion Assay

**Figure 10:**
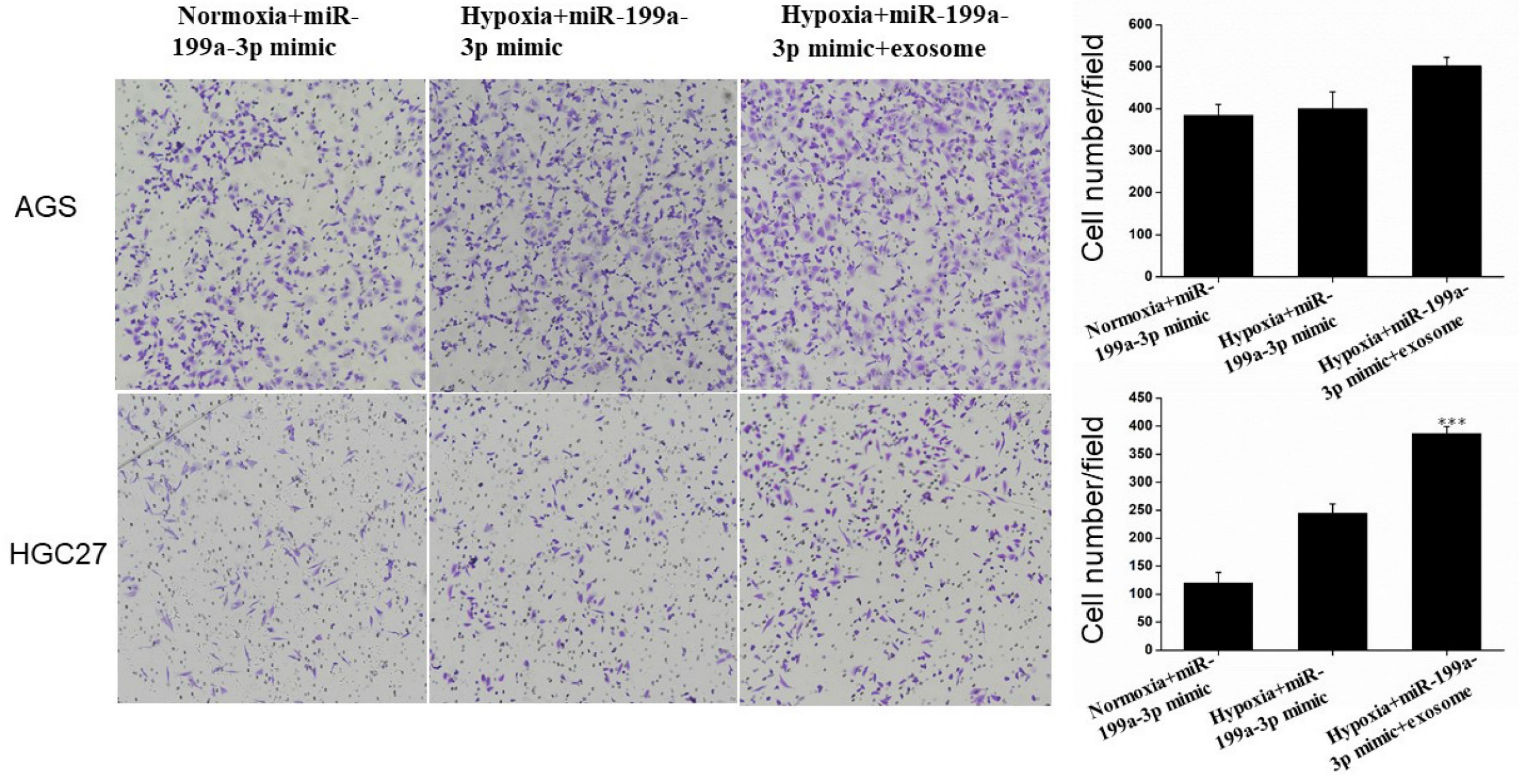
Transfection of exosomes and miR-199a-3p under hypoxic conditions enhances the ability of AGS and HGC27 cells to invade.

Under hypoxic conditions, miR-199a-3p promotes the ability of AGS and HGC27 cells to invade. Transfection of exosomes and miR-199a-3p under hypoxic conditions also enhances the ability of tumor cells to invade, this effect being more pronounced for HGC27 cells (Fig. 10).

### MAP3K4 overexpression with clinicopathologic features and prognosis

**Figure 11:**
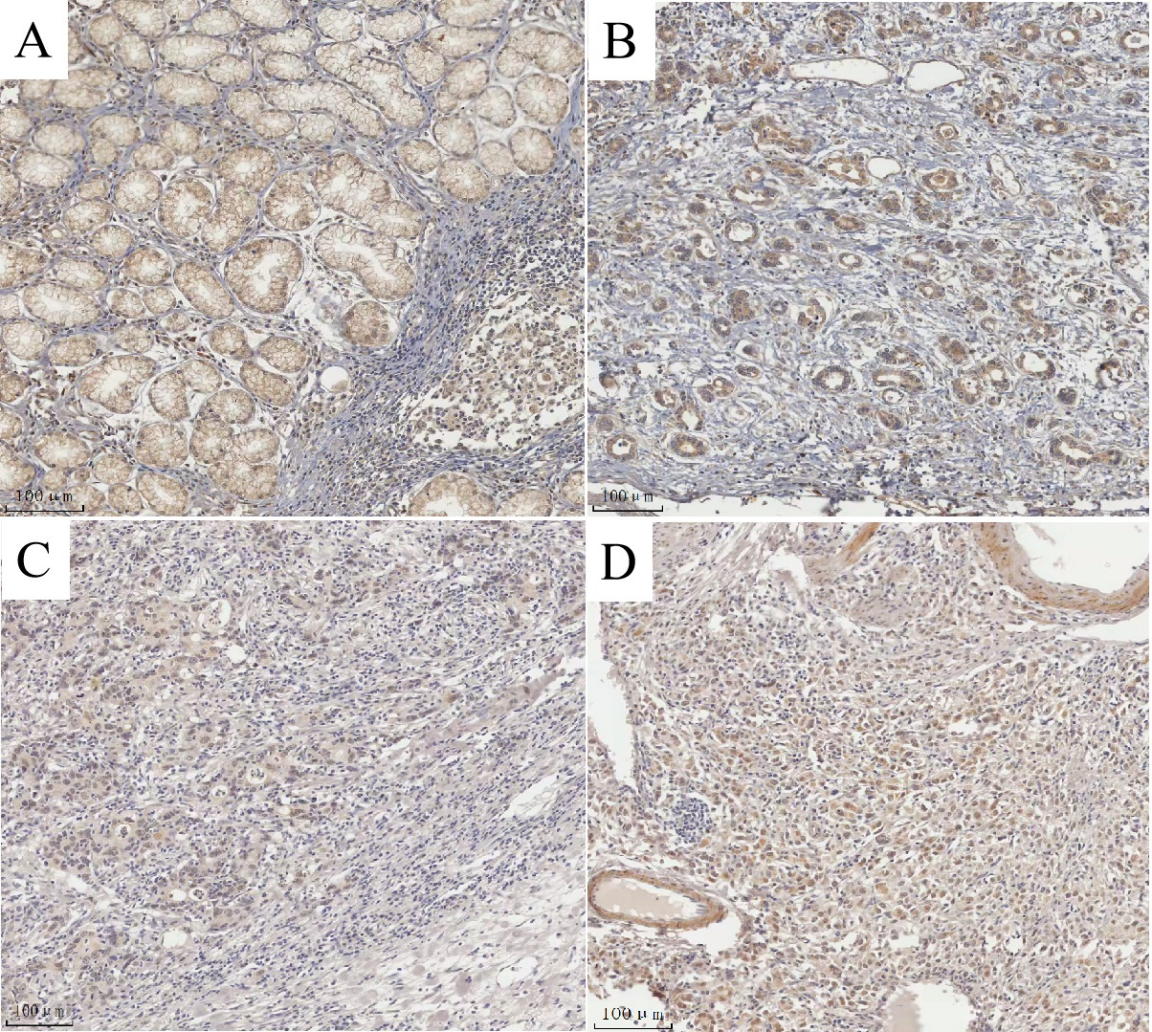
MAP3K4 expression determined in gastric cancer lesions and noncancerous tissues by IHC. A: MAP3K4 was weakly expressed in noncancerous tissues. B, C: MAP3K4 was mainly localized in the cytoplasm of cancer cells, MAP3K4 was highly expressed in moderately differentiated adenocarcinoma. D: MAP3K4 was highly expressed in poorly differentiated adenocarcinoma.

**Figure 12:**
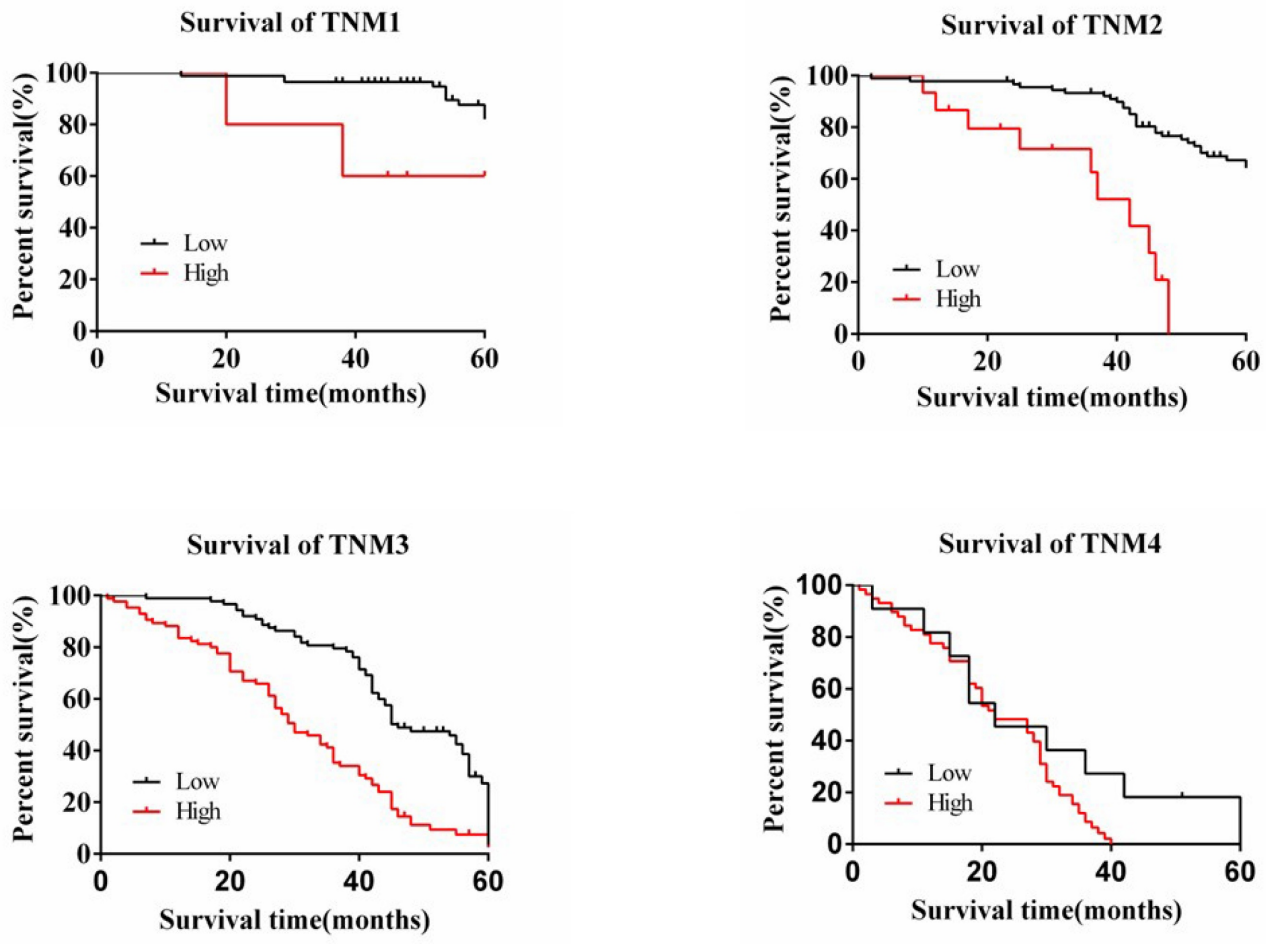
The relationship between the 5-year survival rate of patients with the expression of MAP3K4 in stages I, II, III and IV. The 5-year survival rate of patients with stages I, II and III gastric cancer and weak MAP3K4 expression was significantly greater than that of those with strong MAP3K4 expression. In patients with stage IV disease, MAP3K4 expression is not correlated with 5-year survival.

The degree of expression of MAP3K4 in 436 paraffin-fixed samples of human gastric cancer tissue was assessed by immunohistochemical analysis. All cancer patients received routine chemotherapy after surgery and no radiation. The mean follow-up time was 60 months by the end of December 2008. Survival time was measured from the date of surgery to the follow-up deadline or date of death, which mainly resulted from carcinoma recurrence or metastasis. The Review Board of our Hospital Ethics Committee approved the study, and informed consent was obtained from each participant before data collection. All experiments were performed in accordance with relevant guidelines and regulations of our Hospital Ethics Committee. MAP3K4 was strongly expressed in paracancerous non-neoplastic gastric mucosa and in 36.92% (161/436) of samples of gastric cancer tissue, mainly in the cytoplasm (Fig. 11). Intensity of expression was correlated with tumor size, Lauren classification, depth of invasion, lymph node metastasis, stage of lymph node metastasis, distant metastasis and TNM stage (Table 1). The 5-year survival rate of patients with strong MAP3K4 expression was significantly lower than that of those with weak MAP3K4 expression (P <0.0001). The 5-year survival rate of patients with stages I, II and III gastric cancer and weak MAP3K4 expression was significantly greater than that of those with strong MAP3K4 expression (P =0.0102; P < 0.0001; P < 0.0001, respectively) (Fig. 12). In patients with stage IV disease, MAP3K4 expression is not correlated with 5-year survival (P=0.0577) (Fig. 12). Analysis of independent prognostic factors indicated that MAP3K4 expression, depth of invasion, lymph node metastasis and TNM stage are independent prognostic factors for gastric cancer.

### Association among expression of miR-199a-3p and MAP3K4

Two hundred and ten gastric cancer cases had a low expression of miR-199a-3p and MAP3K4 simultaneously. Ninety-nine gastric cancer cases had a high expression of miR-199a-3p and MAP3K4 at the same time. There was a significant correlation between miR-199a-3p and MAP3K4 (χ^2^=57.31, P<0.001; Table 2).

## Discussion

Johnstone et al. first reported the existence of exosomes, which are cellular vesicles secreted by reticulocytes, in 1987, 10. Exosomes are nano-sized extracellular vesicles and are released by almost all types of cells promoting tumorigenesis, growth, angiogenesis, immune escape and tumor drug resistance. Exosomes play an important role in development and progression of gastric cancer 11-13. Exosomes of diameter 50-100 nm have been observed in human gastric cell line GES-1 and gastric cancer cell lines AGS and HGC-27 under both normal and hypoxic conditions. We detected expression of exosome markers CD9, CD63 and CD81 in the extracted exosomes by label-free proteomics analysis, proving that we had extracted exosomal components.

Exosomes contain many nucleic acids, including miRNA, mRNA, lncRNA, tRNA, snRNA, snoRNA and circRNA; these may regulate gene expression and potentially serve as biomarkers 14. Intercellular interactions mediated by exosomes in the microenvironment play an important role in progression of cancer. Hypoxia is a key precancerous feature of the tumor microenvironment. It impacts the release and composition of exosomes, inducing more aggressive cancer phenotypes. Hypoxic tumor-derived non-coding RNAs in exosomes are basic to regulation of tumor biology and remodeling of the tumor microenvironment 15. We found that expression of miR-199a-3p in exosomes of AGS and HGC-27 cells was significantly stronger under hypoxic conditions than under normal conditions; however, there was no significant difference in expression of miR-199a-3p in GES-1 exosomes between the two states. These findings indicate that hypoxia can induce expression of miR-199a-3p in exosomes of tumor cell lines.

Tumor-derived exosomal miRNA has been proven to promote tumor formation, progression and metastasis by regulating tumor angiogenesis, epithelial-mesenchymal transition and macrophage polarization 16-19. MiR-199a-3p is strongly expressed in gastric cancer tissue 20-22. We found that hypoxia promotes expression of miR-199a-3p in AGS and HGC-27 cells. Under hypoxic conditions, miR-199a-3p promotes invasion and the ability of cells to migrate under hypoxic conditions, this being enhanced by exosomes.

MicroRNAs, single-stranded RNA with 20-24 nucleotides, play biological roles by regulating transcription and/or translation of protein-coding genes 23. To explore the possible regulatory pathway by which miR-199a-3p promotes cancer invasion and migration, we performed label-free proteomic analysis and bioinformatics of exosomes and found that miR-199a-3p may regulate the MAPK signaling pathway. Firefly and Renilla luciferases indicated that MAP3K4 may be the target gene of miR-199a-3p. Expression of MAP3K4 protein increased 24 h after transfection with 199a-3p inhibitor. MAP3K4 is strongly expressed in breast cancer samples 24 and MAP3K4 mutations have been found in 29.2% of tumor microenvironment immune types stage II/III gastric 25. We found that MAP3K4 is strongly expressed in 36.92% (161/436) of gastric cancer samples, the strength of expression being correlated with tumor size, Lauren classification, depth of invasion, lymph node metastasis, lymph node metastasis stage, distant metastasis, TNM stage and prognosis.

MiR-199a-3p 7 and MAP3K4 are strongly expressed in gastric cancer samples, and are correlated with the patient's prognosis. Under hypoxic conditions, we found that exosomal mir-199a-3p may promote tumor invasion and migration by regulating the expression of its downstream molecule MAP3K4 by bioinformatics analysis, firefly and renilla luciferases, western blotting. However, further in vitro and in vivo evidence is needed to confirm this.

## Figures and Tables

**Table 1:** Relationship of MAP3K4 expression with pathological parameters of tumor (number/%)

Clinical parameters	MAP3K4					
	Low	High	t/χ^2^	*P*		
**Age(yrs)**	57.23±11.49	62.10±12.56	4.139	<0.01		
**Gender**			0.873	0.350		
Male	199(64.0%)	112(36.0%)				
Female	74(59.2%)	51(40.8%)				
**Location**			5.40	0.067		
Proximal	27(49.1%)	28(50.9%)				
Middle	102(62.6%)	61(37.4%)				
Distal	144(66.1%)	74(33.9%)				
**Size**			20.84	<0.01		
<5cm	183(71.5%)	73(28.5%)				
≥5cm	90(50.0%)	90(50.0%)				
**Lauren classification**			138.213		<0.01	
Intestinal	199(89.2%)	24(10.8%)				
Diffuse	74(34.7%)	139(65.3%)				
**Histology classification**			1.192		0.755	
Papillary adenocarcinoma	9(56.2%)	7(43.8%)				
Tubular adenocarcinoma	208(63.8%)	118(36.2%)				
Mucinous adenocarcinoma	16(55.2%)	13(44.8%)				
Signet-ring cell carcinoma	40(61.5%)	25(38.5%)				
**Histologic differentiation**			4.197		0.241	
Well	11(84.6%)	2(15.4%)				
Moderately	81(63.3%)	47(36.7%)				
Poorly	179(61.1%)	114(38.9%)				
Others	2(100.0%)	0(0%)				
**Invasion depth**			52.147		<0.01	
T1	53(93.0%)	4(7.0%)				
T2	83(76.1%)	26(23.9%)				
T3	129(52.9%)	115(47.1%)				
T4	8(30.8%)	18(37.4%)				
**TNM Stages**			136.786		<0.01	
Ⅰ	85(94.4%)	5(5.6%)				
Ⅱ	89(85.6%)	15(14.4%)				
Ⅲ	88(50.9%)	85(49.1%)				
Ⅳ	11(15.9%)	58(84.1%)				
**Lymphatic metastasis**			70.06	<0.01		
No	145(87.3%)	21(12.7%)				
Yes	128(47.4%)	142(52.6%)				
**Regional lymph nodes**			97.251	<0.01		
PN0	145(87.3%)	21(12.7%)				
PN1	83(61.0%)	53(39.0%)				
PN2	39(39.4%)	60(60.6%)				
PN3	6(17.1%)	29(82.9%)				
**Distant metastasis**			64.728	<0.01		
No	263(70.1%)	112(29.9%)				
Yes	10(16.4%)	51(83.6%)				
						

**Table 2:** The relationship between the of expression of miR-199a-3p and MAP3K4 in paraffin-fixed gastric cancer tissue.

	MAP3K4		χ^2^	P
miR-199a-3p	Low	High		
Low	210	64		
High	63	99	57.312	<0.001
